# The Role of STAT3 in Non-Small Cell Lung Cancer

**DOI:** 10.3390/cancers6020708

**Published:** 2014-03-26

**Authors:** Daijiro Harada, Nagio Takigawa, Katsuyuki Kiura

**Affiliations:** 1 Department of Thoracic Oncology, NHO Shikoku Cancer Center, 160 Minami-Umemoto-cho, Matsuyama 791-0280, Japan; E-Mail: h401068@yahoo.co.jp; 2 Department of General Internal Medicine 4, Kawasaki Medical School, 2-1-80 Nakasange, Kita-ku, Okayama 700-8505, Japan; 3 Department of Allergy and Respiratory Medicine, Okayama University Hospital, 2-5-1 Shikata-cho, Kita-ku, Okayama 700-8558, Japan; E-Mail: kkiura@md.okayama-u.ac.jp

**Keywords:** signal transducer and activator of transcription 3, Janus kinase 2, epidermal growth factor receptor, non-small cell lung cancer, drug resistance

## Abstract

Persistent phosphorylation of signal transducer and activator of transcription 3 (STAT3) has been demonstrated in 22%~65% of non-small cell lung cancers (NSCLC). STAT3 activation is mediated by receptor tyrosine kinases, such as epidermal growth factor receptor (EGFR) and MET, cytokine receptors, such as IL-6, and non-receptor kinases, such as Src. Overexpression of total or phosphorylated STAT3 in resected NSCLC leads to poor prognosis. In a preclinical study, overexpression of STAT3 was correlated with chemoresistance and radioresistance in NSCLC cells. Here, we review the role of STAT3 and the mechanisms of treatment resistance in malignant diseases, especially NSCLC. As STAT3 is a critical mediator of the oncogenic effects of EGFR mutations, we discuss STAT3 pathways in EGFR-mutated NSCLC, referring to mechanisms of EGFR tyrosine kinase inhibitor resistance.

## 1. Introduction

Signal transducer and activator of transcription 3 (STAT3) is an important signaling mediator in malignant diseases and is persistently activated in 22%~65% of non-small cell lung cancers (NSCLC) [1,2,3]. STAT3 activation by cytokines, such as interleukin-6 (IL-6), is mediated through the Janus family kinases (JAK) or Src [4]. STAT3 is also involved in one of the epidermal growth factor receptor (EGFR) downstream pathways [5]. Several JAK/STAT3 inhibitors suppressed activation of STAT3 and showed anticancer and antiangiogenic effects *in vitro* and *in vivo*. Some have been introduced into clinical trials for solid tumors [6,7,8]. Here, we discuss the role of STAT3 in malignant diseases, especially NSCLC. In addition, we discuss the STAT3 pathways in EGFR-mutated NSCLC. Although STATs have numerous functions in immunoregulation, they have been well reviewed and summarized [9,10]. Therefore, in this review we focus on the oncogenic effects of STAT3.

## 2. Characteristics of STATs

A variety of signal pathways concerning carcinogenesis, malignant transformation, tumor progression and metastasis have been discovered. The RAS/mitogen-activated protein kinase (MAPK) and phosphatidylinositol 3-kinase (PI3K)/AKT pathways have been investigated extensively in oncogenic signaling [11,12,13,14]. One of the more recently recognized oncogenic signaling pathways involves the STAT, which are cytoplasmic proteins with Src homology 2 (SH2) domains. They function as transcription factors responding to cytokines and growth factors in normal cells. This family of proteins consists of seven members: STAT1–4, STAT6, and the closely related STAT5a and STAT5b proteins. The STATs are activated by phosphorylation of a conserved tyrosine residue. Subsequently, they dimerize, translocate to nucleus, bind to DNA and activate the target genes [15,16]. Cytokines and growth factors which phosphorylate the tyrosine residue include IL-6, IL-2, IL-7, epidermal growth factor (EGF), fibroblast growth factor, and platelet-derived growth factor. Soon after growth factors or cytokines bind to their corresponding receptors, intrinsic receptor tyrosine kinases or receptor-associated kinases including JAK or Src tyrosine kinase are activated [17,18]. Subsequently, the tyrosine kinases phosphorylate the receptor to provide docking sites for the recruitment of monomeric STATs. Once they have been adopted, STATs themselves become substrates for tyrosine phosphorylation. Non-receptor tyrosine kinases, such as Src and Bcr-Abl, can phosphorylate STATs independently of receptor activation. Phosphorylated (p) STATs dimerize and translocate to the nucleus, and the dimers regulate gene expression [18]. STAT1, STAT3, and STAT5 are also phosphorylated on a serine residue within their COOH terminus; this phosphorylation is dispensable for dimerization, nuclear translocation, and DNA binding, but is required for maximal transcriptional activity of certain genes [19]. These proteins have dual roles as cytoplasmic signaling proteins and as nuclear transcription factors that activate a diverse set of genes implicated in malignant progression. Since cell cycle is also regulated by the target genes of STATs, the activation promotes cell growth and induces transformation to malignant phenotype.

## 3. STAT3 in Tumor Cell Proliferation

STAT3 has been reported to be associated with oncogenesis and resistance to chemotherapy. While STAT activation is strictly controlled in normal cells and plays a key role in multiple cellular functions, such as cell growth and differentiation, metabolism, hematopoiesis, host defense and immunoregulation, the durable activation of tyrosine kinases in malignant tumor causes constitutive activation of STATs, particularly STAT3 and STAT5 [10,18,20]. Following the activation of STATs, expressions of genes which regulate cancer progression processes including uncontrolled proliferation, apoptosis resistance, sustained angiogenesis, and evasion of immune surveillance would change [18,20,21,22]. Recent evidence suggests a crucial role for STAT family proteins, especially STAT3, in selectively inducing and maintaining a pro-carcinogenic inflammatory microenvironment, both at the initiation of malignant transformation and during cancer progression [23,24,25,26,27,28,29,30,31,32]. Several reports showed that inhibition of STAT3 suppressed the growth of cancer cells and enhanced the sensitivity to anticancer agents in multiple types of cancer [33,34,35,36,37,38,39]. Therefore, STAT3 has been considered a potential target for anticancer therapy.

## 4. Prognostic Impact of STAT3 Activation in NSCLC

To explore the relationship between STAT3 or pSTAT3 expression and its prognostic function on NSCLC, several studies evaluated the clinical significance in patients to determine their effects on tumor angiogenesis and proliferation [3,40]. Jiang *et al*. showed the positive rate of pSTAT3 expression in patients with lymph node metastasis was 78.8% (41/52), which was significantly higher than that in NSCLC patients with non-lymph node metastasis (54.7%, 41/75) using immunohistochemical staining among the 127 cases of p-STAT3 immunoreactivity, was significantly correlated with sex (*p =* 0.004), smoking history (*p =* 0.006), EGFR mutation status (*p =* 0.003), clinical stage (*p =* 0.034), and lymph node metastasis (*p =* 0.009) [3]. Xu *et al*. performed a meta-analysis including a total of 17 retrospective trials and found high STAT3 or pSTAT3 expression was a strong predictor of poor prognosis among patients with NSCLC [40]. Zhao *et al*. reported that NSCLC patients with high pJAK2 expression who underwent surgery had a significantly poorer overall survival rate compared with those with low pJAK2 expression [41]. They also found that microvessel density (MVD) was higher in NSCLC samples with high pJAK2 and pSTAT3 expression, and that patients with high MVD had poor survival rates. These data suggests that high pSTAT3 expression is a strong predictor of poor prognosis in patients with NSCLC. However, it should be confirmed by a large prospective study with long-term follow-up.

## 5. STAT3 in Primary Resistance to Treatment

Primary resistance to treatment includes (1) cytotoxic chemotherapy (2) radiotherapy, and (3) targeted therapy. First, Barre *et al*. reviewed that the STAT3 oncogenic pathway was associated with intrinsic resistance to chemotherapeutic agents in several malignancies [42,43]. In NSCLC cells, overexpression of STAT3 mRNA was related with cisplatin-resistance [42,43]. Silencing STAT3 by siRNA led the resistant cells more sensitive to cytotoxic agents such as paclitaxel, doxorubicin and cisplatin [44,45,46]. Secondary, several studies have suggested that the upregulation of STAT3 directly confers a drug-resistant phenotype, such as resistance to ultraviolet radiation-induced apoptosis [32,47]. You *et al*. showed that ionizing radiation induces phosphorylation of JAK2 and STAT3 and higher levels of STAT3 were found to be accumulated in the nucleus of radioresistant NSCLC cells. They showed niclosamide, a potent STAT3 inhibitor that disrupted STAT3 transcriptional activity by blocking its phosphorylation and nuclear translocation, alone or in combination with radiotherapy overcame radioresistance in xenograft models [48]. Sun *et al*. reported that a small molecule inhibitor of JAK2 (TG101209) was able to inhibit survivin, reduce pSTAT3, and sensitize lung cancer cells to radiation *in vitro* [49]. Thirdly, Looyenga *et al*. demonstrated that STAT3 activity was unaffected by the TKIs such as sunitinib or crizotinib in spite of PDGFR or MET amplified NSCLC cells, respectively [2]. They also showed the treatment of NSCLC cell lines with ruxolitinib, a JAK1/2 selective inhibitor which has shown promising results in phase I/II study for patients with myelofibrosis, inhibited the cell growth in soft agar and in xenograft assays [2]. Inhibition of STAT3 was an effective strategy for overcoming resistance to a dual inhibitor of PI3K and mTOR [50]. These suggest that persistent STAT3 may be leading to primary resistance to targeted therapies. Thus, the JAK2/STAT3 pathway seemed to be associated with primary resistance to treatments, including cytotoxic chemotherapy, radiotherapy and targeted therapy.

## 6. Activation of STAT3 Independent of EGFR Downstream Pathways

There are a variety of pro-inflammatory cytokines and growth factors causing EGFR-independent STAT3 activation. Some tumor cell lines need constitutive activation of STAT3 in order to survive [20,51]. STAT3 was activated by IL-6 in myeloma and prostate cancer cells [24,52,53], hepatocyte growth factor (HGF) and its receptor (c-MET) in leiomyosarcoma and breast cancer cells [54,55], and Src in breast cancer and melanoma cells [54,56].

**Figure 1:**
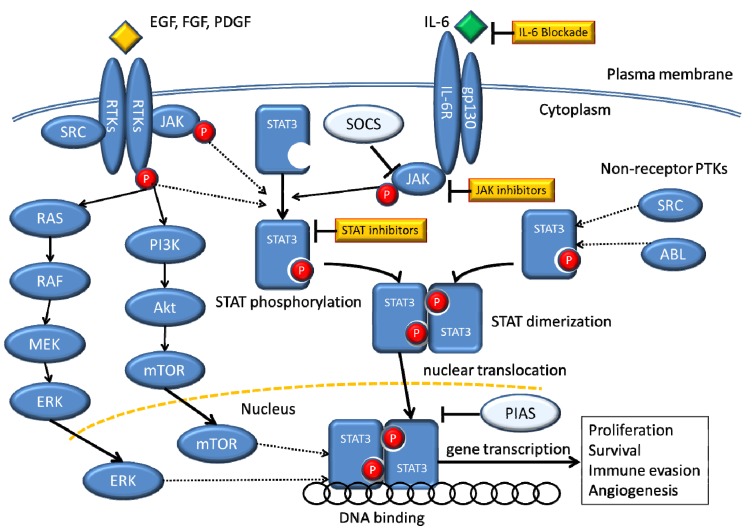
Signaling pathways that converge on STAT3.

In NSCLC, STAT3 is an oncogene that is expressed in alveolar type II epithelial cells [21,57]. Li *et al*. showed that persistent STAT3 activation induced pulmonary tumorigenesis in STAT3 transgenic mice [58]. The activation of STAT3 by receptor tyrosine kinases, such as EGFR and MET, cytokine receptors, such as IL-6 receptor, and non-receptor kinases, such as Src, regulates survival pathways in certain NSCLC cells (Figure 1) [59]. Zimmer *et al*. suggested that STAT3 activity contributes to the carcinogenic potential of NSCLC independently of EGFR mutations [1]. Looyenga *et al*. demonstrated that STAT3 is constitutively activated in human NSCLC samples and in a variety of NSCLC lines independent of activating KRAS, EGFR and PDGFR mutations or MET amplification. They further showed that STAT3 is activated via autocrine signaling by IL-6 family ligands and genetic or pharmacologic inhibition of the gp130/JAK2 signaling pathway disrupts activation of STAT3 [2]. These data suggest that autocrine inflammatory cytokine signaling via JAK2 is the primary driver of STAT3 activation in NSCLC cell lines and in patient tissues.

## 7. STAT3 in EGFR Signaling Pathways

Somatic mutations have been discovered in the EGFR tyrosine kinase in a subset of NSCLC patients [60,61,62,63,64]. These mutations strongly sensitize the cancer cells to the growth suppressive effects of EGFR-TKI, leading to clinical responses [60,61,63,64]. PI3K/AKT, RAS/MAPK, and STAT3 are three major downstream pathways activated by EGFR phosphorylation [65].

STATs are activated by receptor and non-receptor tyrosine kinases through several mechanisms. The receptor-associated Janus family tyrosine kinases (JAKs) are activated by cytokine-receptor binding through cross-phosphorylation, and then the activated JAKs phosphorylate tyrosine residues within the receptor molecules, which become docking elements for cytoplasmic STATs. STATs are subsequently phosphorylated on a single tyrosine residue and form dimers through reciprocal interaction between the phosphotyrosine of one STAT molecule and the Src homology-2 (SH2) domain of another. Dimerized STATs translocate to the nucleus to induce target gene transcription by binding to specific regulatory elements. Receptors with intrinsic tyrosine kinase activity (RTKs), including the platelet-derived growth factor receptor (PDGFR) and the epidermal growth factor receptor (EGFR), may directly activate STATs without activation of JAKs. STATs can be phosphorylated by constitutively active non-receptor protein tyrosine kinases (PTKs), such as Src and Bcr-Abl. Abbreviation: PTK, protein tyrosine kinase; IL-6, interleukin-6; P, phosphorus; SOCS, suppressor of cytokine signaling; PIAS, protein inhibitor of activated STAT.

STAT3 has also been reported as a critical mediator of the oncogenic effects caused by EGFR mutations [66]. Several NSCLC cell lines contain constitutively active STAT3 [59]. Non-transformed epithelial cells engineered to express various NSCLC-associated EGFR mutations had elevated levels of activated STAT5 and STAT3 [63]. Greulich *et al*. reported that STAT3 was activated by various EGFR mutations, including the exon 19 in-frame deletion or the exon 21 L858R point mutation, and may contribute to the oncogenic effects of these mutations in fibroblasts and human lung cancer cells [67]. The mechanism whereby mutant EGFR drives STAT3 activation is indirect, as it requires upregulation of the cytokine IL-6, which activated the gp130/JAK pathway [68] (Figure 1). There has been no strong data suggesting that tyrosine receptor kinases such as EGFR, MET or PDGFR directly drive constitutive STAT3 activation in NSCLC, so far.

## 8. Relationship between STAT3 and Acquired Resistance to EGFR-TKI

First-generation EGFR-TKIs (gefitinib and erlotinib) inhibit EGFR signaling cascade by reversible binding to the adenosine triphosphate (ATP) binding site of the EGFR [69]. Although initial responses to reversible EGFR-TKIs in most patients with EGFR activating mutations were observed, almost all patients acquired resistance to these agents via diverse mechanisms [70]. A secondary T790M mutation (leading to acquired resistance) in exon 20 of EGFR occurred in approximately half of the patients [71]. 

Kim *et al*. demonstrated a role for the IL-6R/JAK1/STAT3 signaling pathway, leading to STAT3 activation, in *de novo* resistance to irreversible EGFR-TKIs, such as afatinib, in NSCLC cells with EGFR T790M [72]. Because STAT3 is not only a downstream effector molecule of IL-6 but also a transcription factor for IL-6 activation [73], autocrine IL-6 production by afatinib could be explained by the activation of a positive feedback in the IL-6/STAT3 axis [72]. It was also suggested that activation of NF-κB stimulated production of IL-6, and that the IL-6-stimulated activation of STAT3 is a mechanism of resistance to EGFR-TKI [74]. As paracrine effect of IL-6 secreted by fibroblasts, IL-6 receptor signal was activated and resistance to afatinib in cancer cells was shown [72]. The tumor microenvironment producing soluble factors such as cytokines and growth factors would help tumor cells to escape from anticancer therapy-induced apoptosis [72,75]. The IL-6 in the tumor microenvironment is known to induce resistance to anticancer agents in malignant diseases [24,76,77]. Because IL-6 is mainly derived from fibroblasts, macrophages and endothelial cells [78,79], high IL-6 levels in the tumor microenvironment might reduce the tumor response to afatinib [72]. 

Furthermore, serum IL-6 levels were elevated in patients with lung cancer in comparison to healthy individuals [80,81]. Under such circumstance, the combination of an IL-6R/JAK1/STAT3 pathway inhibitor with EGFR-TKI may be effective in NSCLC patients with acquired resistance to EGFR-TKI [72]. We established an erlotinib-resistant lung cancer cell line, PC-9/ER3, by continuous exposure of PC-9 cells to erlotinib. The PC-9/ER3 cells did not carry the T790M mutation or MET gene amplification. Although STAT3 was activated in the resistant cell line, inhibition of JAK2 rather than STAT3 restored sensitivity of PC-9/ER3 cells to erlotinib. Thus, activation of STAT3 in this cell line did not directly cause the resistance to EGFR-TKI in NSCLC with activating EGFR mutations. As IL-6 levels in PC-9/ER3 cells were similar to those in PC-9 cells, activation of STAT3 did not seem to be dependent on the IL-6R/JAK1/STAT3 axis [82].

Li *et al*. reported that inhibition of EGFR by erlotinib induced STAT3 phosphorylation on Tyr705 together with increased Bcl2/Bcl-XL at both the mRNA and protein levels in human lung cancer cell lines [83]. They suggested that erlotinib-enhanced phosphorylation of STAT3 may occur from suppression of PTPMeg2 expression, as PTPMeg2 is a physiological STAT3 phosphatase that can directly dephosphorylate STAT3 on Tyr705. Niclosamide, a STAT3 inhibitor, was able to restore sensitivity to erlotinib *in vitro* and *in vivo* [83]. These findings suggested that blocking the PTPMeg2/STAT3/Bcl2/Bcl-XL survival signaling pathway in human lung cancer may provide a novel strategy to overcome acquired resistance to EGFR-TKI.

## 9. STAT3 as a Target for Cancer Therapy

One approach to inhibiting STAT signaling is to block the upstream tyrosine kinases that are responsible for their activation. For example, small molecule inhibitors of JAK, Src, Bcr-Abl, and EGFR have all been shown to block STAT3 signaling and induce tumor cell apoptosis (Figure 1) [24,56,84,85,86]. A STAT3 decoy which comprises a 15 bp double-stranded oligonucleotide competes between the endogenous *cis*-elements within the regulatory regions of target genes and the exogenously added molecules mimicking the specific *cis*-elements [87]. The decoy which corresponds to the STAT3 response element in the c-fos promoter and binds competitively to STAT3 has been shown to inhibit the growth of human lung cancer [88,89]. In addition, a human phase 0 study of a STAT3 decoy oligonucleotide suggested that it might yield effective therapeutic agent for reducing STAT3 target genes and inhibiting tumor growth [90]. Although studies regarding JAK inhibitors in malignant diseases began in the mid-1990s, more potent JAK inhibitors have been developed since the discovery of the *JAK2* mutation in myeloproliferative disorders. These inhibitors were extremely effective for treatment of myeloproliferative preclinical models, leading to clinical trials [7]. For solid tumors, the role of JAK inhibition was examined in models of IL-6-driven breast, ovarian, and prostate cancers using the JAK1/2 inhibitor AZD1480, which led to the suppression of tumor growth [91]. There have been clinical trials testing STAT3 inhibitors, including upstream TKIs for solid tumors, such as lung cancers (Table 1). 

**Table 1:** STAT3 inhibitors tested in clinical trials for solid tumors.

Drug	Target	Indication	Status
Siltuximab (CNTO-328)	IL-6 (monoclonal antibody)	Ovarian, pancreatic, colorectal, head and neck, and lung cancer	Phase I Phase II
Ruxolitinib (INCB018424)	JAK1/2	Chronic myeloproliferative disorders, leukemia, myelodysplastic syndrome, myeloproliferative neoplasms, unspecified childhood solid tumor	Phase I
		Metastatic cancer, pancreatic cancer	Phase I
AZD1480	JAK1/2	Advanced solid tumor, hepatocellular carcinomaEGFR and/or ROS mutant lung cancer, metastatic lung cancer, gastric cancer	Phase I
INCB047986	JAK	Advanced solid tumor, Hodgkin's lymphoma, non-Hodgkin's lymphoma	Phase I
OPB-31121	JAK2 and gp130	Advanced solid tumor	Phase I
WP1066	JAK2 and gp130	Glioblastoma multiforme, melanoma	Phase I
OPB-51602	STAT3 phosphorylation	Advanced cancer	Phase I
SAR302503	JAK	Solid malignancy	Phase I

Several agents targeting the IL-6R/JAK/STAT3 signaling pathway, such as siltuximab (IL-6-neutralizing monoclonal antibody [92]), OPB-31121 (small molecule inhibitor JAK2 [93]), and AZD1480 (small molecule inhibitor of JAK [8,94]), have been clinically developed and may be suitable candidates for future combination therapy with EGFR-TKIs. At present ruxolitinib [2] is only JAK1/2 selective inhibitors that has been approved for use by the FDA to treat JAK2-dependent malignancies such as myelofibrosis. Recently, microRNAs were reported to regulate the activity state of STAT3 or to be regulated by STAT3 activation in tumors [95,96,97,98]. The microRNA upregulated by STAT3 activation might be associated with the mechanisms of cancer invasion, migration and resistance to anti-cancer agents including cytotoxic or targeted therapies. In particular, miR-337-3p directly targeted to STAT3 and sensitized NSCLC cells to paclitaxel [99]. The treatment of anticancer agents with microRNA down-regulating STAT3 might be investigated.

## 10. Conclusions

STAT3 signaling is an important pathway in a variety of malignant diseases, including NSCLC. JAK/STAT3 inhibitors may be effective for treatment of NSCLC irrespective of the EGFR mutation status. We need to elucidate the mechanism(s) of STAT3 activation from autocrine, paracrine or stromal sources of ligand. STAT3 activation by stromal cells may contribute to the oncogenic effects of the NSCLC cells. Subsequently, the unknown role(s) of STAT3 in therapeutic resistance should be resolved. Finally, the most promising avenues for use of JAK or STAT inhibitors in a clinical context, for example, whether they would work as monotherapy or only as combination therapy should be explored. More translational and clinical trials are required to clarify the potential roles of STAT3 inhibitors in the treatment of NSCLC.
